# The Role of Implicit and Explicit Staff Attitudes in the Use of Coercive Measures in Psychiatry

**DOI:** 10.3389/fpsyt.2021.699446

**Published:** 2021-06-18

**Authors:** Angelika Vandamme, Alexandre Wullschleger, Amelie Garbe, Celline Cole, Andreas Heinz, Felix Bermpohl, Juliane Mielau, Lieselotte Mahler, Christiane Montag

**Affiliations:** ^1^Department of Psychiatry and Psychotherapy, Berlin Institute of Health, Charité Universitätsmedizin Berlin, Freie Universität Berlin, Corporate Member of Freie Universität Berlin, Humboldt-Universität zu Berlin, Berlin, Germany; ^2^Division of Adult Psychiatry, Department of Psychiatry, Geneva University Hospitals, Geneva, Switzerland; ^3^Department of Psychiatry, Clinics in the Theodor-Wenzel-Werk, Berlin, Germany

**Keywords:** staff attitudes, implicit attitudes, coercion, psychiatry, GNAT

## Abstract

Many determinants leading to the use of different coercive measures in psychiatry have been widely studied and it seems that staff attitudes play a crucial role when it comes to the decision-making process about using coercion. However, research results about staff attitudes and their role in the use of coercive measures are inconsistent. This might be due to a focus on self-report studies asking for explicit answers, which involves the risk of bias. This study aimed to expand research on this topic by examining the impact of explicit and implicit staff attitudes on the use of coercive measures in clinical practice. In addition, the influence of gender, profession (nurses, psychiatrists), and years of professional experience as well as their influence on staff attitudes were examined. An adaption of the implicit association measure, the *Go/No-Go Association Task (GNAT)*, with the target category *coercion* and distracter stimuli describing *work load*, as well as the explicit questionnaire *Staff Attitudes to Coercion Scale (SACS)* was completed by staff (*N* = 149) on 13 acute psychiatric units in 6 hospitals. Data on coercive measures as well as the total number of treated cases for each unit was collected. Results showed that there was no association between staff's implicit and explicit attitudes toward coercion, and neither measure was correlated with the local frequency of coercive measures. ANOVAs showed a significant difference of the GNAT result for the factor gender (*F* = 9.32, *p* = 0.003), demonstrating a higher tendency to justify coercion among female staff members (M = −0.23, SD = ±0.35) compared to their male colleagues (M = −0.41, SD = ±0.31). For the SACS, a significant difference was found for the factor profession (*F* = 7.58, *p* = 0.007), with nurses (M = 2.79, SD = ±1.40) showing a more positive attitude to the use of coercion than psychiatrists (M = 2.15, SD = ±1.11). No significant associations were found regarding the extent of professional experience. Results indicate a complex interaction between implicit and explicit decision-making processes dependent on specific contexts. We propose future research to include primers for more context-related outcomes. Furthermore, differences in gender suggest a need to direct attention toward occupational safety and possible feelings of anxiety in the workplace, especially for female staff members.

## Introduction

Despite continuous international efforts to reduce coercion in acute psychiatric inpatient care, measures such as restraint, seclusion or compulsory medication remain regularly used interventions (1, 2). Supporting patients in regaining their health while maintaining a safe and secure environment on psychiatric units regularly leads to staff members facing difficult decision-making processes. Although, coercive measures have proven to be lifesaving in certain situations (3) they also yield the risk of detrimental consequences for patients and may result in additional and long-lasting mental health conditions such as PTSD (4). Coercive measures are considered serious violations of an individual's right to self-determination and personal freedom and therefore need to be reduced to those situations in which no other measures can save the patient's life or prevent severe harm to the patient herself or others (5). In situations where psychiatric staff decides a coercive measure is indicated, it is crucially important that this decision is reviewed and authorized by the responsible judicial authority. In Berlin (Germany) where this study was conducted, the legislative background for coercive measures “the law for help and safety precautions in case of psychiatric diseases” (PsychKG) (6) regulates the application of such measures in a very strict manner to ensure coercion is applied solely as last resort. This means, coercive measures such as seclusion or restraint always need to be approved by court for a determined time frame and the patient has to be under continuous medical observation. In addition, every coercive measure should subsequently be reflected together with the patient to identify strategies to prevent further coercion during treatment. In recent years, efforts to promote human rights in the field of mental health have increased substantially and patients' human rights, empowerment, user participation, and the reduction of coercion in mental health care have become a center of attention in health care policies worldwide (7). Therefore, determinants which lead to the use of different coercive measures have been widely studied in the last decade. Cultural (8) and organizational climate (9) have been suggested to have a decisive impact on the use of coercive measures in clinical inpatient settings, as well as the quality of the therapeutic relationship between patients and staff members (10, 11). Furthermore, patient (10, 12) and staff factors such as gender (13), stature (14) and experience (15) have been identified as relevant criteria regarding the use of coercive measures. Although, research on this topic shows inconsistent results, one important staff factor seems to be the attitude of individual staff members toward these kind of methods (14). According to one of the most common definitions, attitudes can be described as “learned predispositions to think, feel and behave in a specific manner to a certain object” (16). This definition is known as the three-component view of attitude and includes affective (feeling and emotions), cognitive (believes, thoughts, attributes) as well as behavioral (past behavior and experiences) aspects. Moreover, attitudes comprise both, contents that is accessible to the conscious mind and can be verbally, explicitly expressed, but also the implicit imprints of past experiences that might be not or not correctly identifiable by introspection, but nevertheless can guide behavior (17).

The first studies on the topic of attitudes toward coercive measures focused exclusively on seclusion and were conducted between 1978 and the end of the 1990's. These studies indicated an explicit positive staff attitude toward coercive measures (18, 19) and showed that these interventions were considered an appropriate tool and part of routine clinical practice (20, 21). During the last two decades, the number of research projects on staff attitudes toward coercion increased and results, especially from the field of nursing science, show that a slightly more negative attitude developed over time (22, 23). Further, individual staff factors and their connection to explicit attitudes toward coercive measures have been investigated. Gender seems to be the most reported staff factor but results remain inconsistent (23). Husum et al. (24) reported that women rated coercion marginally less as treatment compared to men. In addition, Falkum and Førde (25) found female psychiatrists to be less in favor of paternalism, advocate for more patient autonomy and engage in deeper moral deliberation about coercive measures. However, other studies did not find a correlation between staff's gender and their attitudes (26). The profession has also been suggested to be associated with staff attitudes on coercion. Some scholars found that nurses tend to approve coercive measures more than psychiatrists (27, 28). However, Mötteli et al. (26) report the opposite. Less research has been conducted on correlations between staff attitudes toward coercion and work experience and results for this factor are inconclusive (23, 26).

To the authors knowledge, all previous research on staff attitudes toward coercion has focused solely on its explicit dimension but never on its implicit processes. This might be due to a methodological focus on self-report studies asking directly for experiences or perspectives and thus acquiring deliberate answers on a given topic. These deliberate answers involve the risk of bias, mainly due to social desirability. This risk is particularly prevalent when it comes to socially controversial issues and is therefore a highly relevant factor in researching staff attitudes toward coercive measures in psychiatry.

In contrast to explicit measures which capture more elaborate and conscious goals, implicit measures seem to prompt earlier, spontaneous and affective processes (29). Therefore, Greenwald et al. (30) developed a computerized test based on reaction times, the implicit association test (IAT), in order to assess the content of implicit memory through spontaneous and intuitive responses (31). The test performed successfully on different topics such as race or stigma toward people with mental health conditions (32, 33) but has never been adopted to the question of implicit staff attitudes toward coercion in psychiatric inpatient care. Furthermore, there are only few studies which examine the relation between implicit attitudes and actual behavior (34) and, to the best of our knowledge, no previous study has been conducted on the research question at hand.

The aim of this study is to investigate implicit staff attitudes in psychiatric inpatient care using a modification of the IAT, namely the GNAT (short for Go/No Go Association Task) (35), and to compare explicit and implicit attitudes regarding their predictive value for the use of coercive measures on psychiatric units. We expect both explicit and implicit staff attitudes to have an influence on the decision-making process and thus the actual performance of coercive measures. Furthermore, we aimed to gain more clarity on the influence of the staff's factors gender, profession, and work experience on their attitudes toward coercion. It was hypothesized (1) that explicit attitudes would reflect implicit attitudes, (2) that both implicit staff attitudes as well as explicit staff attitudes show an association with the number of coercive measures on the respective units and (3) the staff factors gender, profession and work experience would show an association with implicit as well as explicit staff attitudes.

## Method

The present study was part of a larger RCT, primarily designed to examine effects of post-coercive review sessions on coercion-related outcomes (ClinicalTrials.gov ID NCT03512925) financed by the German Ministry of Health. This analysis focused on the attitudes of staff members toward coercive measures. To prevent confounding effects due to a more profound engagement with coercion and its consequences, the present study was conducted at the beginning of data collection.

### Participants and Recruitment

Participants (*N* = 149, *n* = 93 nurses, *n* = 56 psychiatrists, 77 female, 72 male) were recruited in six different psychiatric clinics in Berlin, Germany, on 13 acute inpatient units. All participating units function as mandatory health care providers for a defined catchment area and are conducting coercive measures regularly. The heads of the participating clinics approached staff members to participate and motivated the staff in team meetings as well as during the shifts research assistants were present to conduct the test.

### Study Measures

#### Go/No-Go Association Task

**Figure 1:**
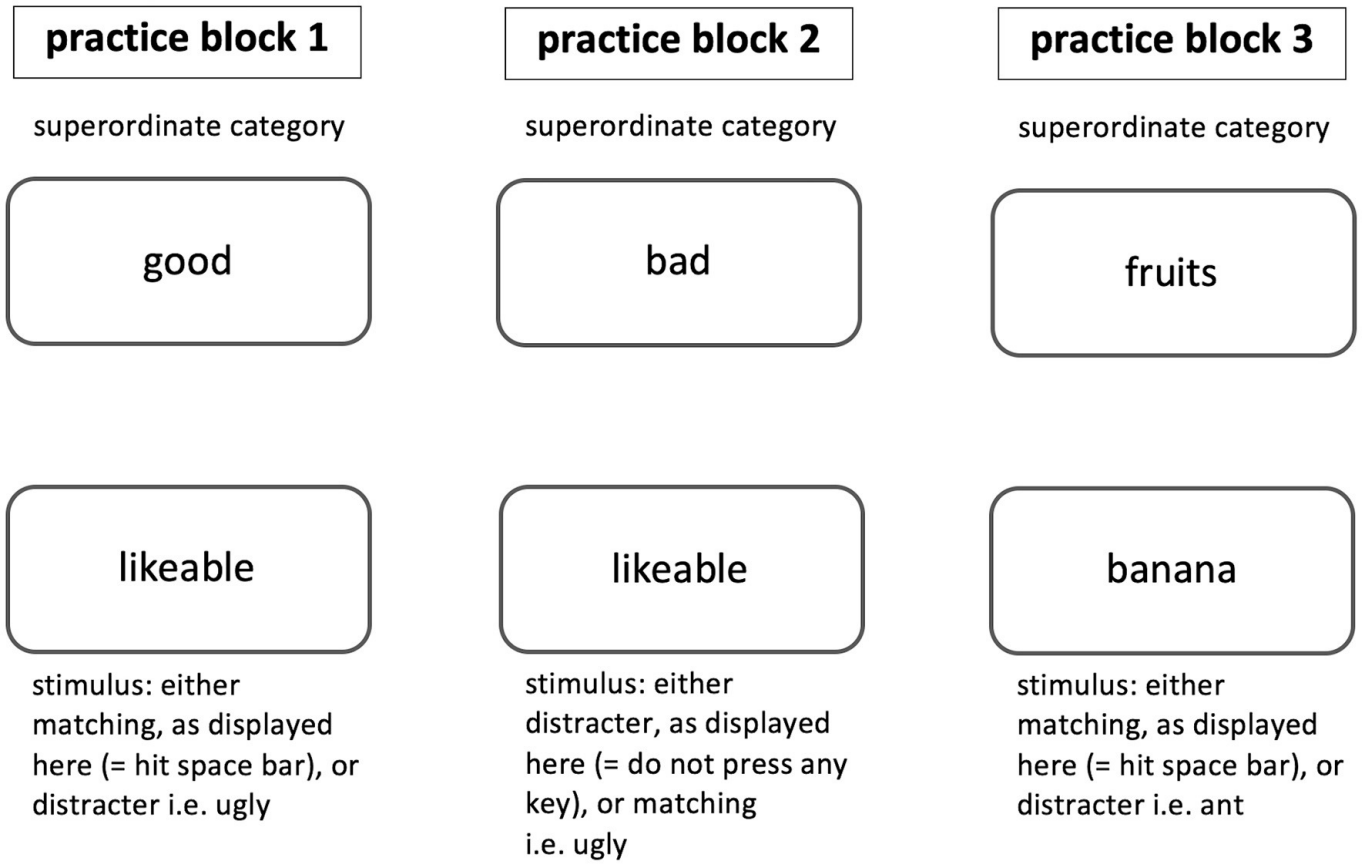
Practice blocks as they may appear in a GNAT. The superordinate category stays the same throughout the practice block, whereas, stimuli change after a deadline is reached or the space bar was pressed.

The Go/No Go Association Task (GNAT) is a computerized implicit association measure regularly used in social psychological research. The GNAT was developed by Nosek and Banaji (35) as an enhancement of the Implicit Association Test (IAT). In the GNAT, stimuli have to be classified into superordinate categories, while speed of classification is being measured in order to assess the strength of automatic association in memory. Compared to the broadly used IAT, the GNAT requires only one target category (i.e., “fruits”) and two attribute dimensions (i.e., “good” and “bad”), which allows the investigation of implicit targets with no corresponding category. The test usually consists of five blocks. The first three blocks serve as training and answers are not included in the subsequent analysis. Figure 1 displays an example of the three practice blocks as they may appear in a GNAT. Each trial of the training condition shows one stimulus either of matching (i.e., “banana”) or distracter type (i.e., category bugs: “ant”) and the superordinate target category (i.e., “fruits”) on the screen. Participants are assigned to discriminate between the displayed stimuli and to react accordingly: In case the displayed stimulus belongs to the target category, the correct response is to press the space-bar of the keyboard. If the displayed stimulus does not belong to the displayed superordinate target category, the participants are asked not to press any key at all. A response deadline for each trial is set determining when the next stimulus appears on the screen.

**Figure 2:**
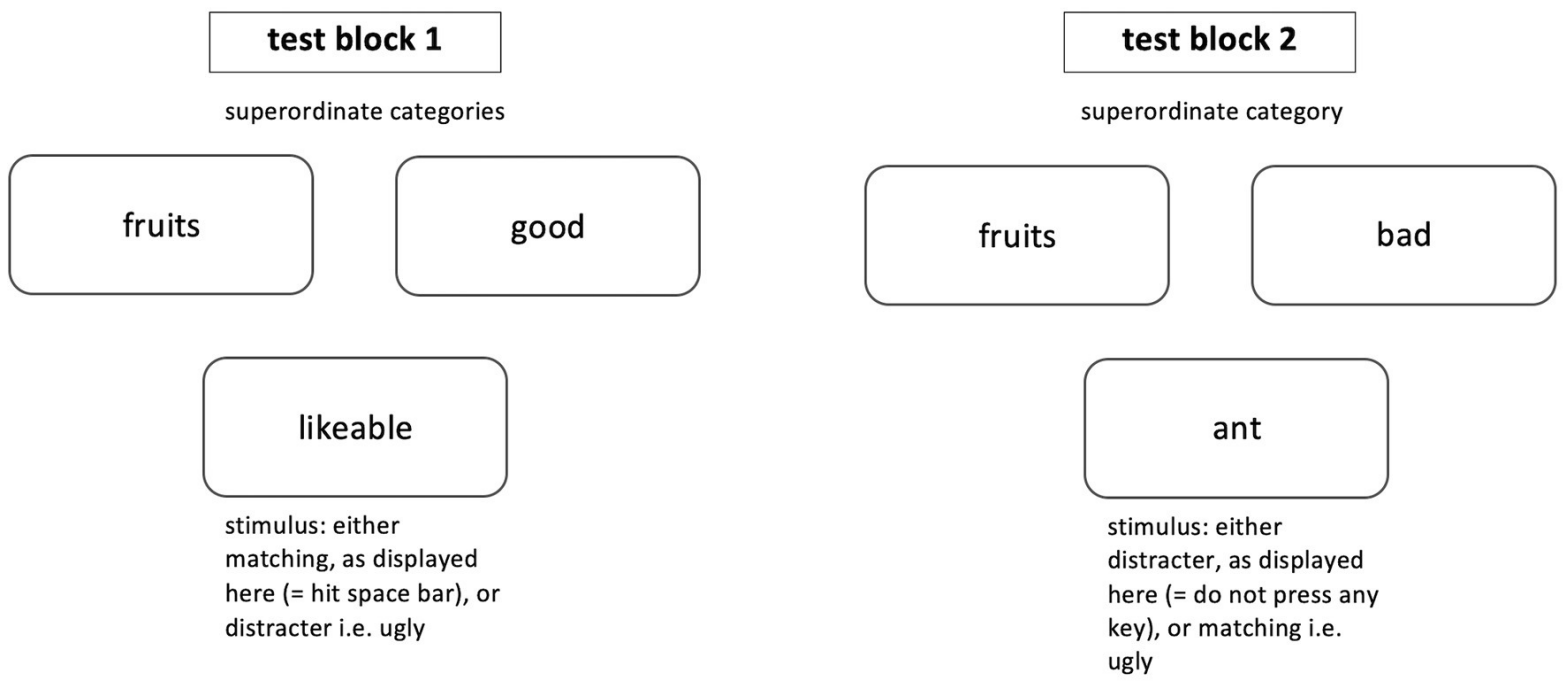
Test blocks as they may appear in a GNAT. The superordinate categories stay the same throughout one test block, whereas stimuli change after a deadline is reached or the space bar was pressed.

The next two blocks serve as test blocks and answers are included in the analysis. Figure 2 displays an example of the two test blocks as they may appear in a GNAT. Each trial of the test condition shows one stimulus, again, either of matching (i.e., “tasty,” “banana”) or distracter (i.e., “ugly,” “ant”) type and this time two superordinate target categories (i.e., “good” and “fruits”) appear on screen. Participants are instructed to discriminate between the displayed stimuli and to react accordingly: In case the displayed stimulus belongs to one of the target categories, the correct response is to press the space-bar of the keyboard. If the displayed stimulus does not belong to one of the displayed superordinate target categories, the participants are asked not to press any key at all and wait until the deadline is reached and the next stimulus appears on the screen. A response deadline for each trial is set determining when the next stimulus appears on the screen.

Response time for trials displaying target stimuli are recorded. The degree of association between the target category and one of the two attribute dimensions is characterized by faster responses in one condition compared to the other. For this study, the GNAT was adapted as originally published by Nosek and Banaji (35) in experiment 3 of the paper.

##### Conceptualizing the GNAT for This Study

**Figure 3:**
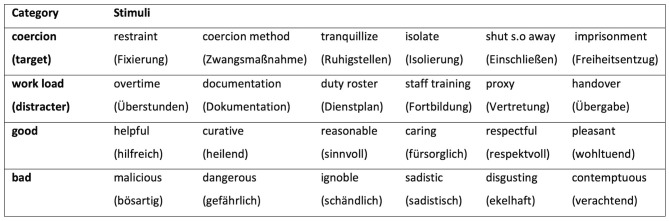
Categories and stimuli used in the GNAT.

For this study, a GNAT was developed to assess the strength of association between the target category “coercion” and a descriptor, namely two poles of the attribute dimensions “good” vs. “bad.” Piloting for the GNAT stimuli was conducted to ensure the used stimuli were sufficiently distinctive and intuitive to be quickly categorized and word length was similar for all used words. Twenty staff members of an acute psychiatric unit were asked to rate six different lists, each consisting of 18 words (lists: good, bad, therapy methods, work load, freedom, and coercion methods). Participants were asked to rate those words using the three dimensions of the self-assessment-manikin (SAM) (36): valence, arousal, and dominance. Categories and stimuli were selected by considering the mean, standard deviation and deviation from neutrality, in order to find emotionally potent words for the attribute dimensions “good” and “bad,” as well as for the target category “coercion.” As recommended by Nosek and Banaji (35), the most neutral words were chosen for the distracter category which in our case were the words of the list “work load.” Chosen categories and stimuli are displayed in Figure 3.

**Figure 4:**
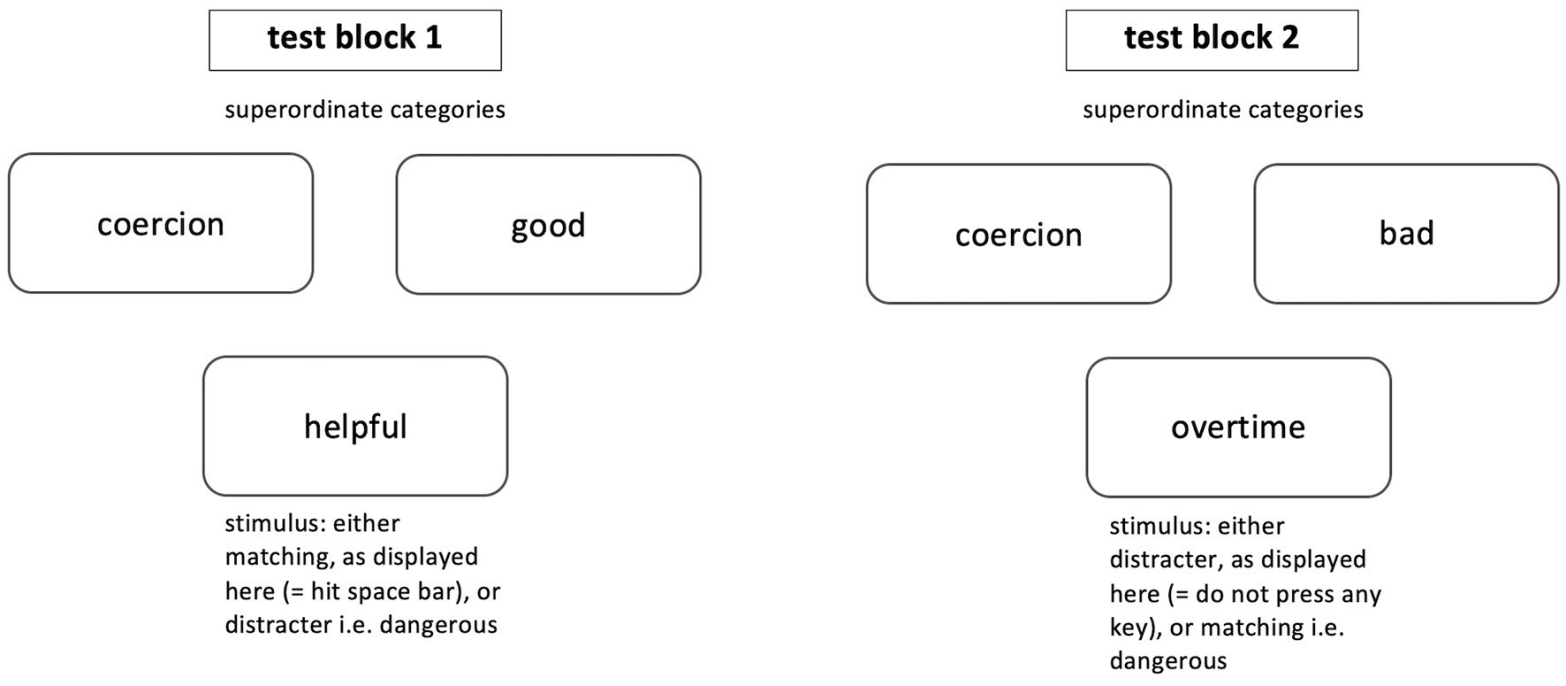
The two critical combined test blocks of the GNAT are displayed. The superordinate categories stay the same throughout one test block, whereas, stimuli change after a deadline is reached or the space bar was pressed.

The test consisted of five blocks. The first three blocks were 30-trial randomized single categorization blocks, each with 15 target or descriptor and 15 distracter stimuli. The stimuli were presented in a random order and counterbalanced, which served as practice, so subjects could attune to the procedure, stimuli and task at hand. The next two critical combined test blocks included stimuli from target, descriptor and distracter categories at the same time and are displayed in Figure 4. Coercion served as target category, either good or bad was the descriptor category (depending on the block) and the respective other was the distracter. The two test blocks were also randomized including 63 trials. Target and distracter stimuli were randomized and counterbalanced.

Stimuli in all five blocks were presented for 850 ms, with an inter-stimulus interval of 150 ms for all five blocks, as recommended by Nosek and Banaji (35). Feedback on accuracy was given for 100 ms after each trial by a green “O” when the answer was correct or a red “X” in case of an incorrect answer.

#### Staff Attitude to Coercion Scale

Data on explicit attitudes toward coercion was collected by using the Staff Attitude to Coercion Scale (SACS), a questionnaire assessing how individual mental health care professionals perceive attitudes toward coercion among all staff members as a group (37). The questionnaire consists of 15 items on three dimensions of attitudes: (1) coercion as offending (critical attitude), (2) coercion as care and security (pragmatic attitude) and (3) coercion as treatment (positive attitude) and shows good and stable psychometric properties. Participants are asked to rate how strongly they agree or disagree with a given statement on a five-point Likert scale ranging from “disagree strongly” (1) to neutral (3) to “agree strongly” (5). Scores for each subscale are calculated by building the sum of the corresponding items of each subscale. Furthermore, an overall SACS score can be calculated by reversing the items of the “coercion as offending” scale and finally creating a total sum of all 15 items. A higher total value indicates a more positive attitude toward coercion.

#### Frequency of Coercive Intervention and Number of Treated Cases

Statistical data of performed coercive measures included each act of restraint (mechanical restriction of a patient's freedom of movement using special straps) or seclusion (locked isolation room in which the patient can move freely but is unable to leave) conducted within 1 year. In addition, the total number of treated cases for the same year was obtained from each participating clinic. These statistics were part of the mandatory annual reporting to governmental institutions (Berlin Senate) and provided by the heads of departments.

## Procedure

The authors assert that all procedures contributing to this work comply with the ethical standards of the relevant national and institutional committees on human experimentation and with the Helsinki Declaration of 1975, as revised in 2008. All procedures involving subjects were approved by the ethics committee of the Charité Universitätsmedizin Berlin (ID: EA1/158/17). Written informed consent was obtained from all participants.

First, participants were asked to fill out a question form which inquired gender (m, f) and years of professional work experience in six groups (< 5, 5–10, 10–15, 15–20, 20–25, >25 years). Next, the developed offline PsychoPy (38) computerized GNAT was presented using a 13″ laptop screen. Completing the GNAT took ~5–7 min.

Last, the SACS questionnaire was completed by the participants. Although, the chance of deliberately faking implicit associations is reportedly very low, this order of proceedings was chosen to avoid priming on attitudes toward coercion by completing the SACS first and therefore potentially influencing the results of the GNAT.

## Data Analyses

All statistical analyses were conducted using the integrated development environment RStudio. The threshold for statistical significance was set to *p* < 0.05. Reported correlations are Spearman correlations. Assumption of homogeneity of variance and assumption of normality were verified by Bartlett tests and Shapiro-Wilk tests, respectively. Group comparisons were then conducted using ANOVAs with added contrast analysis where applicable. Due to differences in reporting of coercive measures between hospitals the data was grouped per participating hospital when analyzing the relation between both SACS and GNAT scores and conducted coercive measures.

### GNAT

Scoring and data reduction of the GNAT was conducted following recommendations made by the test developing authors (35, 39) and on the basis of the research by Teachman (40). Error rates for each of the 149 participants were checked and data sets with an error rate exceeding 40% per block were deleted, as well as data sets with more than 30% error rates on the task overall. Cases with more than 10% responses under 300 ms on trials were also removed, leaving 120 datasets for statistical analysis. Since distracter trials are considered noise, only target and descriptor trials of the two critical combined blocks were used for data analysis. Next, single trials with a response under 300 ms were deleted due to the possibility of random answers. The average error rate for the cleaned data sets was 16%. The GNAT D-Score was then calculated for each participant by dividing the difference between the mean reaction times of the two critical combined blocks (coercion & good minus coercion & bad) by the standard deviation of the N latencies. Higher D-scores indicate stronger positive implicit associations toward coercive measures.

### SACS

Eight missings were calculated over all questionnaires and replaced by the global means of the respective answers to the items. The total SACS score was calculated as proposed by Husum et al. (37).

## Results

### Implicit vs. Explicit Attitudes and Coercive Measures in Psychiatric Clinics

Due to low quality of reported data on coercion rates, one clinic unit was removed for this analysis with a total of 104 data sets remaining. GNAT D-Score resulted in an overall mean of −0.31 (SD = 0.34) and SACS with a mean 2.49 (SD = 0.34). Since data on coercive measures was only measurable on a clinic's level, averaged D-Scores and SACS- Scores were obtained for all six clinics' staff members. All means and standard deviations for each variable of every participating clinic are displayed in Table 1.

**Table 1:** Descriptive statistics for D-scores and SACS scores, the number of treated cases, the total number of coercive measures, and the relative frequency of coercion.

**Clinic**	**M (SD) D-score**	**M (SD) SACS**	***n* cases**	***n* coercion**	**Relative frequency of coercion**
Clinic A	−0.34 (±0.34)	2.36 (±1.78)	1,115	33	0.03
Clinic B	−0.30 (±0.40)	2.60 (±1.69)	1,580	514	0.33
Clinic C	−0.33 (±0.35)	2.58 (±1.03)	1,220	98	0.08
Clinic D	−0.35 (±0.33)	2.53 (±1.02)	462	192	0.42
Clinic E	−0.25 (±0.37)	2.63 (±0.97)	1,063	291	0.27
Clinic F	−0.41 (±0.27)	2.2 (±1.53)	550	82	0.15

*M mean, SD standard deviation, n-cases total number of cases, n-coercion total number of performed coercive measures, relative frequency of coercion ratio of total number of performed coercive measures to total number of cases*.

There was no significant association between the implicit measure GNAT and the explicit measure SACS (r sp = 0.07, *p* = 0.48). The correlation of the D-Score on a clinic's level with the rate of coercive measures in each hospital was not significant (*r* = 0.09, *p* = 0.91). The correlation between the SACS sum score and quantity of coercive measures (*r* = 0.37, *p* = 0.5) indicates a stronger association, yet the z-test on the difference between the two correlations did not reach significance (z = 0.37, *p* = 0.36).

### Differences in Gender, Professions, and Years of Professional Experience

As the following comparisons did not rely on clinic level data, the analysis was conducted for all remaining data sets after data reduction as described above (*n* = 120). Since only 29 participants categorized themselves in the four groups of more than 10 years of work experience, those groups were consolidated, resulting in: group 1 (<5 years, *n* = 64), group 2 (5–10 years, *n* = 27), and group 3 (>10 years, *n* = 29). Means and standard deviations for all three variables (gender, profession, professional experience) for the GNAT and SACS are reported in Table 2.

**Table 2:** Descriptive statistics and group comparisons of implicit (GNAT) and explicit (SACS) measures regarding gender, profession, and professional experience.

	***N***	**M (SD) D-score**	**M (SD) SACS**
Gender		^**^	
Women	62	−0.23 (±0.35)	2.38 (±1.38)
Men	58	−0.41 (±0.31)	2.62 (±1.23)
Profession		^*^	^**^
Nurses	65	−0.25 (±0.38)	2.79 (±1.40)
Psychiatrists	55	−0.39 (±0.29)	2.15 (±1.11)
Professional experience			
Group 1	64	−0.369 (±0.33)	2.37 (±1.25)
Group 2	27	−0.376 (±0.26)	2.63 (±1.06)
Group 3	29	−0.155 (±0.41)	2.65 (±1.64)

*N population size, M D-Score mean D-Score GNAT, SD D-Score standard deviation D-Score GNAT, M SACS mean SACS questionnaire, SD SACS standard deviation SACS questionnaire ANOVA (dependent variable: D-score or SACS, factors: gender, profession, professional age)*,

** *p < 0.01*,

* *p < 0.05*.

#### D-Score

The conducted ANOVA on the GNAT as a dependent variable including all three independent variables yielded a significant effect of gender (*F* = 9.32, *p* = 0.003) with women (M = −0.23, SD = 0.35) showing a significantly higher D-Score than men (M = −0.41, SD = 0.31). Differences in profession also proved to be significant (*F* = 5.88, *p* = 0.017) with nurses (M = −0.25, SD = −0.39) showing a higher D-Score than psychiatrists (M = −0.39, SD = 0.29).

The analysis did not show significant differences for professional experience (*F* = 1.94, *p* = 0.15) between the three age groups (group 1: M = −0.37, SD = 0.33, group 2: M = −0.38, SD = 0.26, group 3: M = −0.16, SD = 0.41). The two directional contrasts investigated proved to be not significant with group 1 < group 2 (*F* = 0.26, *p* = 0.61) and group 2 < group 3 (*F* = 3.62, *p* = 0.06). No interaction effects proved to be significant.

#### SACS

An equivalent ANOVA model for the explicit measure SACS as a dependent variable using all three independent variables yielded significant differences for the profession (*F* = 7.58, *p* = 0.007), nurses (M = 2.79, SD = 1.40) showing higher values than psychiatrists (M = 2.15, SD = 1.11). Both gender (*F* = 0.82, *p* = 0.37; women: M = 2.38, SD = 1.38, men: M = 2.62, SD = 1.23) and professional experience (*F* = 0.40, *p* = 0.67) did not show significant differences on the SACS (group 1: M = 2.37, SD = 1.25, group 2: M = 1.63, SD = 1.06, group 3: M = 2.65, SD = 1.64). No interaction effects proved to be significant.

## Discussion

Using the GNAT and SACS as measuring instruments, implicit, and explicit staff attitudes toward coercion in psychiatric care were examined for the strength of their association. Furthermore, the relation between staff attitudes and the corresponding occurrence rate of restraint and seclusion was examined across six different psychiatric clinics in Berlin, Germany. In addition, the individual staff factors profession, gender, and professional experience were analyzed regarding their impact on implicit and explicit attitudes toward coercive measures.

No correlation between the implicit measure GNAT and the explicit questionnaire SACS was found. This result may lead to the assumption that both methods measure different constructs (29). Moreover, neuroimaging studies found distinct neurological mechanisms for automatic vs. explicit processes using functional magnetic resonance imaging (41, 42), suggesting that implicit techniques target spontaneous and affective processes (29), whereas, explicit techniques evoke a controlled and conscious answer and thus representing different constructs. Explicit attitudes in particular are subject to transformations by interpersonal and group dynamics, cultural norms, or by only partially related motivations like the wish for justification.

The hypothesis that both implicit and explicit staff attitudes show an association with the number of coercive measures on the respective unit, was not supported by the analysis. Correlations between D-Scores and the frequency of coercive measures on a clinic's level did not reach significance. On a descriptive level, the correlation between the SACS and coercive measures turned out to be stronger, but did not reach significance either.

Previous research from different fields has been trying to link implicit and explicit attitudes to actual behavior (43–45), with moderate success. Until today, it seems difficult to explain the gap between people's attitudes and actual behavior (46). Meissner et al. (46) suggested that associations, as measured by the GNAT, could be too unspecific to unambiguously relate to and account for a particular behavior in a specific situation. Hence, the authors see the assessment of attitudes as a difficulty that is independent of a certain context whereas mental representation of attitudes refer to a specific context. A proposed model by Perugini and Prestwich (47) supports this explanatory approach. The assumption postulates that priming can increase (assimilation effect) or decrease (contrast effect) the likelihood of a person performing a correspondent action depending on the direction and strength of the specific association between a concept and its valence. An interesting approach for future research on coercion in psychiatry might be using case vignettes as primers for a certain context prior to implicit measures. Using adaptations of other implicit association measures, such as the propositional evaluation paradigm (PEP) (48), could be a feasible way to take this specific situational context into account. This test allows for the assessment of more complex propositions, by using full priming statements which have to be categorized in “true” or “false.”

A possible explanation for our results on the connection between implicit and explicit staff attitudes and the performance of coercive measures may lie in the complex interaction between implicit and explicit attitudes. The available literature suggests that the systems might be activated and exert their influence in various ways (49). Following Strack und Deutsch (50) and their reflective impulsive model (RIM), behavior is shaped through the interaction of a reflective (explicit), and an impulsive (implicit) system. Both systems contribute to a behavioral outcome. However, if the two systems activate opposing schemes like the implicit rejection of coercion and an explicit approval to solve a threatening situation at the same time, the result might be conflicting. In solving this conflict, specific circumstances of a situation rather than attitudes determine actual behavior (49).

Furthermore, the role of situational moderators and the influence of cognitive capacity have been discussed scientifically (51). Full cognitive capacity is associated with deliberate, explicit attitudes, whereas, reduced cognitive capacity decreases the influence of reflective processes on judgements and consequently gives more room for impulsive, implicit attitudes (52). In light of the considerably different threatening scenarios in which coercive measures in psychiatry are used, staff members' full cognitive capacities might be altered by intercurrent stressors hindering the process of decision making (explicit attitude). Consequently, impulsive processes might occasionally guide behavior (implicit attitude).

Intragroup dynamics might have a strong impact on explicit attitudes of staff. Opinions, attitudes and behavior of each member of a group are shaped by others within the group through a state of interdependence (53). This means, individuals can take a strong position within the group (i.e., alpha = leader) and influence explicit attitudes and behavior (i.e., pro coercion), as described by Schindler (54) in his rank dynamic model. Staff members might experience aversion toward coercive measures on an implicit level, but fail to screen for appropriate alternatives to address a threatening situation e.g., due to the perceived dominance of the alpha person, but also due to staff shortage or other structural conditions and thus support coercive measures on an explicit level.

However, far beyond these theoretical explanations, it must be admitted that possible connections between attitudes and the frequency of coercive measures might have remained undiscovered due to the unexpectedly heterogeneous quality of data on coercive measures obtained from the participating hospitals. For this reason, data was only analyzable on a clinic level, and not on the level of individual units or wards, which drastically reduced the effective sample size used for examining the first hypothesis.

Comparisons of the implicit as well as explicit attitudes between nurses and psychiatrists showed that nurses are more in favor of coercive measures than psychiatrists. These findings support previous research, showing that nurses evaluate seclusion and restraint as a necessary intervention and an essential part of the job (55, 56). Nurses are generally more often and to a higher intensity exposed to patients' wishes, needs and psychopathology (i.e., due to accessibility of the nurses' staff room on the unit). At the same time, nurses are more frequently exposed to patients' aggressions and as a result might experience more fear and might feel the need to maintain the beneficial atmosphere on the unit for all parties. Consequently, nurses appear to consider coercive measures to some extent as care giving (24). Psychiatrists tend to see their patients on a more selective basis (i.e., for unit rounds), partially having more detailed background information. The difference in the quantity and quality of contact to patients may shape the attitudes of the staff and explain the discrepancy between nurses and psychiatrists regarding the acceptance of coercive measures. Furthermore, nurses and psychiatrists tend to have a different educational background. Psychiatrists usually gain a broader knowledge on psychopathology and psychotherapy due to the structure of their studies and training compared to nurses. Thus, psychiatrists might develop a different attitude toward coercive measures. However, longer professional training is not necessarily linked to attitudes rejecting coercive interventions, or vice versa, and further factors like work-related autonomy and self-efficacy as well as peer dynamics should be included in future studies. Methods of preventing coercion should be addressed when conceptualizing training for all professions working in clinical psychiatry, and the establishment of a shared, multi-professional, therapeutic attitude should always be an important goal within a team.

Our results on gender differences indicate that women seem to show a higher acceptance toward the performance of coercive measures on an implicit level compared to men. So far, research on this topic has offered ambivalent results, as Doedens et al. (23) showed in their systematic review. However, it should be noted that gender differences were not confirmed for explicit attitudes in our sample. Findings might implicate that women may experience more fear in threatening situations compared to men, and must apply more self-control to cope with it. Since high self-control can increase the impact of explicit attitudes and decrease the influence of implicit attitudes (57), more attention should be payed to the management of anxiety and occupational safety. This seems especially relevant for female-dominated teams.

Our analysis on professional experience showed no significant effects of years of professional experience neither for implicit nor for explicit attitudes toward coercive measures. Thus, findings on this topic are still inconclusive. Whereas, research by Gandhi et al. (58) showed, that nurses with more work experience maintain a more positive attitude toward restraint, other authors report the opposite (59). The influence of experience of coercive measures on a quantitative level and attitudes toward coercive measures was highlighted by Molewijk et al. (60): Staff agreed to statements, that coercion can be seen as care and security more readily, when they had used those methods regularly, compared to those staff members who had distinctly less experience with such measures. A review conducted by Doedens et al. (23) took individual staff characteristics as well as organizational factors into account and could not pinpoint any trend, although, a feeling of safety seemed to reduce coercive measures. However, since the standard deviation on years of professional experience in our study turned out to be high, we assume that other, not adequately studied characteristics such as personality traits, individual levels of fear, threatening personal experiences in the past or job satisfaction could shape attitudes toward coercion more profoundly than years of professional experience. Thus, decision-making processes and possible associations between staff attitudes and the actual performance of coercive interventions may differ considerably between teams or units and might not be discernible on a hospital level. These factors may be focus of further studies.

### Limitations

Attitudes are formed and influenced by a number of variables (personality, social aspects, and former experiences) (29) which constitutes a challenge to pinpoint the degree of the respective impact of individual variables on the use of coercion. Hence, measures of attitude are still compromised by moderate quality criteria, implicit measures even more than explicit techniques (35, 61, 62). Besides, IAT-related measures target associations which refer to mental connections between words, but fail to express beliefs. As Houwer (63) suggested, implicit evaluation might influence the activation of an association. For example, the expressions “I am good” or “I want to be good” relate in a different way to each other, but both include the concepts “I” and “good.” A strong association between both words does not provide any information on the personal state of one's evaluation. Starting from the above-mentioned limitation regarding data quality on coercive measures, it should be noted that the system of documentation for coercive measures is not yet standardized in Germany. This means, each hospital documents the measures in a different manner. In one hospital it was not traceable, whether restraints were performed in the emergency room during admission or during treatment on the specific unit. Another hospital did not differentiate between units at all and data on coercive measures for the second half of the year was completely missing. Though, this seems to be a broader problem internationally (64), it even severely complicated comparisons between local hospitals that work according to the same legislation, and lead to a lack of statistical power in the present study.

Furthermore, the definition of coercive measures might lack distinctiveness. One hospital claimed not to use seclusion as a method, but advised patients to stay in their room in certain situations, while a staff member would guard the door. In case the patient aimed to leave the room, staff hindered patients, if necessary, by force. The hospital asserted that the door of the room would never be locked and a staff member is approachable at any time. This shows the challenge of defining coercion and the legal limbo mental healthcare finds itself entangled in. Consequently, the collected data in our study may underrepresent incidents of coercion in some clinics while over representing in others cannot be ruled out with adequate certainty.

This was the first pilot application of a newly developed GNAT to assess attitudes toward coercive measures and thus improvements of the method might be needed to generate more precise results. Although, piloting was conducted, some of the chosen words might be imprecise and word length might be too long considering the deadline of the GNAT. In addition, testing was conducted during shifts in an office on the units. Depending on the workload, the situation on the unit and the duration of the shift, concentration might have been poor, and daily events may have impacted results. Testing in separate facilities outside the unit at beginning of a shift would be preferable.

### Conclusions

This study was the first attempt to link staff members' implicit attitudes to the performance of coercive measures in psychiatry. Extensive research in this field is still needed, as staff and contextual factors influencing implicit and explicit attitudes toward coercion are still inconclusive and the psychiatric discipline is requested to draw relevant conclusions for patient and staff management. Although, first studies set ground for an international approach to explore involuntary admissions and the realization coercive measures (65, 66), a standardized definition and documentation of coercive measures nationally and internationally is urgently needed, allowing to conduct research on causative variables and mechanisms which lead to coercion more accurately and to derive implications for clinical practice from future outcomes.

## Data Availability Statement

The raw data supporting the conclusions of this article will be made available by the authors, on reasonable request.

## Ethics Statement

The studies involving human participants were reviewed and approved by Charité Universitätsmedizin Berlin. The patients/participants provided their written informed consent to participate in this study.

## Author Contributions

AV, CM, AW, and LM are responsible for the original study design from which data was taken. AV and AG collected data. AV and CM performed statistical analyses and wrote the draft with theoretical input of JM and AW and reviewing by CC, AH, FB, and LM. All authors contributed to the article and approved the submitted version.

## Conflict of Interest

The authors declare that the research was conducted in the absence of any commercial or financial relationships that could be construed as a potential conflict of interest.
